# Observational Tools Using Video Recordings of Parent-Child Feeding Interactions: A Narrative Review

**DOI:** 10.3390/children9121924

**Published:** 2022-12-08

**Authors:** Gessica D’Angeli, Martina Mesce, Luca Cerniglia, Silvia Cimino

**Affiliations:** 1 Department of Dynamic, Clinical and Health Psychology, Sapienza University of Rome, 00186 Rome, Italy; 2 Faculty of Psychology, International Telematic University Uninettuno, 00186 Rome, Italy

**Keywords:** review, infant observation, parent-child interaction, feeding, video recordings

## Abstract

Current research has confirmed that the quality of the caregiver-child relationship influences the child’s emotional and behavioral development. Play and feeding contexts, for example, are the best contexts to observe mother-child or father-child interaction. The observation of feeding interaction establishes involvement on the part of both parties and identifies relationship characteristics. The purpose of this study is to select and describe the most frequently used observational methods during feeding interactions in the first three years of a child’s life. Instruments that employ video recordings of mealtimes will be detailed to highlight the relevance that specific tools have nowadays. Finally, the SVIA (Scala di Valutazione delle Interazioni Alimentari), a technique for analyzing food interactions by observation that has also been utilized remotely, will be offered. This is intended to provide practitioners and researchers with an overview of tools while also taking into consideration the present scenario in which digital tools are increasingly being employed in health and clinical settings. Furthermore, the purpose of this paper is to review the various observational methods of the parent-child relationship to assist future practitioners and researchers in the field in making an accurate assessment of caregiver-child interaction and selecting a valid tool for the early recognition of problematic relationships and identifying the most appropriate treatment modalities.

## 1. Introduction

Parent-child interactions are essential as they form the basis for the child’s later emotional and cognitive development and serve as a predictor for children’s experiences with the outside world [1]. Parental responsiveness is considered crucial as it is one of the most influential components of child development in several areas, including cognitive, social, and emotional, and has an impact on the likelihood of the onset of psychological problems [2,3].

In recent years, research has focused on the role of mothers and fathers in the adaptive emotional and cognitive development of the child, particularly the role of fathers as protectors and/or risk factors for the development of difficulties in children. In the context of feeding, researchers have mainly focused on mother-child interactions, as mothers are believed to be primarily responsible for feeding children [4]. According to some studies, mothers and fathers do not differ in their level of sensitivity toward their children [5]. However, other research has shown that fathers are more likely than mothers to encourage children to eat [6], but also that fathers have a lower ability to recognize signs of distress in their children compared to mothers and that fathers are less sensitive and attuned to children in the early years of life [7].

Observation of the interaction between parents and children is essential to identify difficulties and strengths in the relationship, as these can have an impact on the child [8]. Many authors have studied the quality of parent-child interaction and parental responsiveness, describing new components of it; in fact, multiple instruments have been developed to assess its several aspects. Indeed, observational-type instruments assessing parent-child interaction cover a wide range of constructs [9].

One of the key features of parent-child interaction observation tools is that they allow the measurement of dyad-level constructs, such as synchrony or reciprocity because they focus on the coordination of behaviors between the child and the parent. This is critical because parent-child synchrony has been shown to be related to children’s later cognitive development [10,11].

The literature shows how important it is to observe relationships, as humans are born with an innate sensitivity to connect with others and share their experiences. According to Trevarthen [12], through intersubjective experience (the ability to adapt subjective control to the subjectivity of the other to communicate), the child begins to attribute meanings to events in the world. Observational methods developed through the Tavistock method of child observation, in which the observer’s role is to immerse himself or herself in the interaction between parent and child.

According to Corcoran & Fischer [13], observational tools of parent-child interaction should be preferred to self-report measures because the latter can be easily distorted by the tendency to respond in socially desirable ways or by the parent’s thoughts and feelings and language skills. Observational instruments, on the other hand, allow assessment of parent-child interaction and behaviors that often occur without awareness, such as the evolution of parent-child behavior over time and its influence on partner behavior [14]. Therefore, they appear to be the most accurate methods for assessing parent-child behaviors, despite disadvantages such as the large time investment they require [15].

The use of reliable and valid measures to assess the quality of parent-child interaction is essential because only in this way can strategies be provided that can identify non-functional care practices and, therefore, make possible interventions that can improve relationships. Indeed, it has been observed that compromised relationships can lead to the development of psychopathological symptoms as children mature [16]. To identify the most appropriate treatment model, effective and timely assessment of problematic caregiver-child interactions is essential [17].

In this paper, we chose to focus on the qualities of interaction during feeding since in early childhood this activity contributes to the construction of reciprocity in exchanges between parents and children [18]. During breastfeeding, for example, there is mutual involvement between mother and child. According to Stern [19], mother and child adapt to each other, and the mother should be able to recognize the child’s signals, such as hunger and satiety. Dyads instinctively adapt their two-way communications according to context and goals, and the study of play and feeding con-texts is important for identifying characteristics of the caregiver-child relationship [20].

If the caregiver-child dyad is not attuned and does not communicate effectively, it will be difficult for the child to use self-regulatory mechanisms, and this may negatively affect the child’s behavior and emotional state in the future [21,22,23]. Studies have shown that prevention and intervention in early childhood of psychopathological risk symptoms can decrease future psychopathological disorders by reducing maladaptive behavioral functioning in children [24]. Observation and assessment of caregiver-child interactions are therefore crucial because they allow not only for making a diagnosis but also for selecting the most appropriate form of intervention.

Observational-type tools have been in use for some time, but the impact of the COVID-19 pandemic on research has brought new opportunities, especially regarding the use of online tools. In fact, while the possibility of using psychoeducational tools and online clinical sessions for parents and families existed before the spread of the pandemic, the advent of COVID-19 introduced greater use of digital tools and allowed for a different management of time and costs due to travel for health care. For example, some research groups, mentioned in this review, had already experimented with the online use of an observation tool in the context of food interaction. The SVIA scale, during the COVID-19 pandemic, began to be administered remotely with satisfactory results.

Considering this background, this review aims to select and compare strategies for assessing caregiver-child feeding interactions. In particular, this review aims to describe tools that employ video recordings of meals to highlight the relevance of this type of specific tool.

## 2. Materials and Methods

### 2.1. Research Strategy

**Figure 1:**
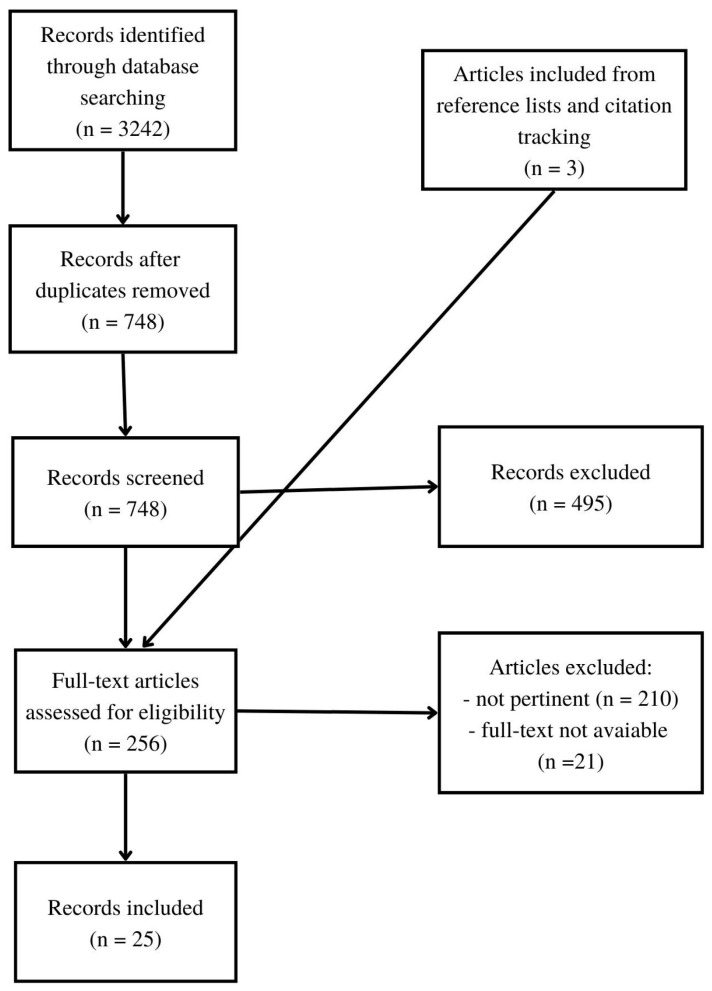
Flowchart.

The objective of this paper was to learn about observational tools currently used via videotaping to assess eating disorders during childhood. The methodological approach used consists of a narrative review [25,26], a form of interpretive-qualitative publication that does not answer a specific question but aims to discuss the state of the art of a given topic and increase the scientific community’s debate on it [27]. Therefore, a non-systematic literature review was conducted in the electronic databases of “PubMed” and “Web of Science” conducted between August and September 2022. No restrictions on the year of publication were used, and the Scopus index was used to check the scientific relevance of the selected articles. Articles that contained any combination of the keywords listed in Table 1 were collected. The search strategy is detailed in Figure 1.

### 2.2. Eligibility Criteria

Instruments were included that (1) were quantitative measures, (2) could be used for infants (age 0–4 weeks) and/or toddlers (age 1 month–1 year) and/or children (age 1–2 years) and/or preschoolers (age 2–6 years), and (3) were used to observe parent-child interaction during mealtime with videotaping. Non-observational instruments (1), which did not investigate parent-child interaction (2), were excluded; in addition, instruments for which a reference describing their psychometric validity could not be found (3) or which had not been evaluated by an experienced external observer (4) were excluded. Articles were selected based on their impact on the scientific community, trying to select them according to the impact factor of the journal in which they were published and according to the number of citations to have high-quality articles. However, because this topic is specific, it was necessary to expand the literature to be considered and it was not always possible to meet the criteria for the number of citations. The decision to include in at page 8, other studies (selected according to the above criteria) that have used the tools in the last 3 years, is because we tried to gather information on the current situation through the most recent studies that had a focus as consistent as possible with the topic of this review, also concerning the use of these tools during the particular period of the COVID-19 pandemic.

## 3. Results

The research conducted identified several studies using mealtime observation tools with the use of video recording. The details are shown in Table 2. Specifically, 11 instruments were identified that met the inclusion criteria described above. All of the selected tools were validated; the characteristics of each instrument were explained in Table 3, which in fact describes the age of the child, the parent(s) involved in the interaction, whether it is necessary to have had training, and the most recent studies that have used that tool.

Below is a brief description of the tools included, grouped according to the following age groups of the child: newborn (ages 0–4 weeks); infant (ages 1 month–1 year) toddler (ages 1–2 years); preschooler (ages 2–6 years).

The Assessment of Mother-Infant Sensitivity (AMIS) [53] scale measures the quality of early mother-infant interactions in a feeding context within the first trimester postpartum. AIMS was developed to measure the quality of early mother-infant feeding interactions in the first 4 months of a baby’s life. Mother-infant interaction during feeding is recorded with a video camera that is placed 2 m from the dyad. The AMIS scale codifies the video of mother-infant interaction, and it consists of 25 items, with possible scores ranging from 1 to 5 points (higher values indicate greater “sensitivity”). Items assess maternal behaviors, infant behaviors, and dyadic behavior. Assessments are made from the observation of 15- to 30-min videos of the of mother-child interaction.

The BATMAN (Bob and Tom’s method for assessing nutrition) [54] is used for toddler to preschooler children and consists of video recordings of real-time observations (from 6 to 41 min) of family meals where the child’s feeding behavior and parental behavior are evaluated. The tool is used by toddlers to preschoolers. A sampling of participants’ behaviors at 10-s intervals is reported. The physical environment (where the interaction takes place), how the child’s behaviors occur (e.g., sucking, refusing food, etc.), the person interacting with the child, the parent’s behaviors (e.g., whether food is offered or whether there is verbal encouragement or discouragement), and how the child responds to the interaction with the parent are also assessed.

The Dyadic Interaction Nomenclature for Eating (DINE) [55] targets children ages 7 months to 12 years (most effective up to preschool age) and consists of videotaping meals to code behavior. Observation of videotaped meals in the home environment assesses the child and parental eating behaviors and child feeding behaviors. DINE codes most behaviors based on presence/absence in 10-s intervals.

The Mealtime Observation Schedule (MOS) [56] has been tailored for observations of child feeding across a wide age range, including the 0–3 age group. MOS analyzes and codes footage of the mealtime observation and assesses both appropriate and disruptive behaviors of the child during the meal, as well as aversive and nonaversive behaviors of the parents. The MOS employs a partial interval time sampling procedure to record the presence of 16 child behaviors and 14 parent behaviors. The MOS is a coding method that differentiates between children with and without feeding difficulties.

Mother-Infant Feeding Tool (MIFT) [52] is for newborns and infants. MIFT encodes videotaped feeding interactions. MIFT is a real-time, coding scheme that was designed to capture the regulatory process of mother-infant feeding interactions. The infant is assessed as regulated or dysregulated in each of 4 areas: muscle tone, physiological indices, behavior and emotions, and physiological indices. Maternal behavior is described with 7 categories of behaviors: monitoring behavior, supportive behavior, verbal behavior, vestibular stimulation, active touching, feeding, and nurturing. The tool allows clinicians to identify and examine specific dysregulation episodes in the baby, such as whether the mother and infant are having difficulty initiating a feeding.

The NCAST developed by Barnard [57], is distinguished into Nursing Child Assessment Feeding Scales and Nursing Child Assessment Teaching Scales. The Teaching (NCATS) and Feeding (NCAFS) scales are organized into six subscales representing 73 and 76 binary items, respectively. Four scales describe parental behavior: (a) sensitivity to cues, (b) response to child distress, (c) promotion of social-emotional growth, and (d) promotion of cognitive growth. Two scales describe the child’s behavior: (a) clarity of cues and (b) the child’s responsiveness to parents. The Teaching Scale assessment targets caregivers and their infants or toddlers between birth and 36 months of age and are ideally conducted in a period between 1 and 5 min; the Feeding Scale assessment targets caregivers and their infants and toddlers between birth and 12 months of age and typically take place over 10 min.

The Parent Child Early Relational Assessment (PCERA) [58] is an observational tool used in children from 0 to 5 years of age that allows the description of the qualities of social-emotional and task-related behavior during mealtime but does not describe the process of interaction between mother and child. The quality of the child-parent relationship is assessed through video-recorded observations of the child interacting with the parent during 4 segments lasting 5 min each that include: feeding, separation/reunion, free play, and structured task. PCERA consists of 65 items each rated on a 5-point behavior rating scale. Each scale assesses an affective and behavioral characteristic that the parent and child bring to the interaction.

Responsiveness to Child Feeding Cues Scale (RCFCS) [59], is used for up to 2 years of age of the child and it is an observational measure of caregivers’ responsiveness to child feeding cues relevant to obesity. General responsiveness during feeding and maternal responsiveness to infant hunger and satiety are assessed using digital recordings; RCFCS was developed to measure the quality of feeding interaction observed during infancy and early childhood. The RCFCS is used to encode videos of feeding interactions. Hunger signals are encoded. The 48 hunger and satiety signals are encoded from the time of food preparation until 1 min after the first bite to assess the infant’s degree of receptivity or disinterest in feeding. Feeding signals are classified as early (e.g., sucking on objects, slowing down or pausing), active (e.g., gaping when food is presented, pushing food away), or late (e.g., crying) to reflect the ways infants are thought to communicate.

The Mealtime Interaction Coding System (MICS) [60] is used for children aged 12 months to 13 years. The MICS is a commonly used measure to assess family mealtime functioning. The MICS uses direct observation and assessment of actual behavior in the family’s natural environment and focuses on interactions and functioning specific to mealtime. The MICS codes six dimensions of family functioning; overall family functioning is the last dimension assessed and provides an overall assessment of the quality of family interactions and functioning during the meal. “Affect Management” and “Interpersonal Involvement” assess the emotional aspects of the family meal. Interpersonal involvement captures the degree to which family members show respect and interest and value for others. “Behavior control” assesses how well the family maintains rules about social behaviors. “Task accomplishment” assesses the structure and organization of the meal and reflects the parents’ ability to have control over the meal. “Communication” assesses the family’s verbal interaction, particularly the quality of communication and appropriateness among different age groups. Finally, “roles” reflect each family member’s behavior patterns and ability to perform expected tasks. Each dimension is rated on a Likert scale from 1 to 7, with scores of 5 or higher considered categorically different (healthy) from those with scores below 5 (unhealthy).

The Lausanne Trilogue Play (LTP) [61] is used for children aged from 2 to 17 years old. LTP is a nonintrusive observational method that provides information about the interactive patterns enacted by the family on various occasions and in different contexts. It allows all possible configurations (mother-child, father-child, mother-father, mother-child, mother-father) to be assessed through a single observation using the following functions: participation, organization, focal attention, and affective contact. The most recent context in which the paradigm has been used is mealtime: the procedure allows family interactions during feeding to be observed and evaluated. The entire procedure is videotaped so that the strengths and weaknesses of the family system and individual dyads can be observed, assessed, and captured.

The Feeding Scale [62] and its Italian version (SVIA) [63] can be used in children from 0 to 3 years old. The Feeding scale comprehensively assesses mother-infant/child interactions and is based on the analysis of a videotaped feeding interaction (during the main meal). The scale consists of 46 items (26 for the mother and 20 for the child) and five subscales. The 5 subscales include (1) Dyadic reciprocity; (2) Dyadic conflict; (3) Chattering and distraction; (4) Struggle for control; and (5) Maternal non-contingency. Mother-infant behaviors are assessed based on 20 min of the videotaped feeding interaction on a 4-point Likert scale according to the frequency and intensity of each of the behaviors. The SVIA is the Italian validation of the Feeding Scale and is composed of 41 Items and divided into 4 subscales: Mother’s Affective State, Interactive Conflict, Infant Feeding, Refusal Behaviors, and Dyad Affective State.

**Table 3:** Characteristics selected tools: Author/Year; Children age; Parent involved; Training; Recent studies.

Tools	Author/Year	Children Age	Parent Involved	Required Training	Studies in the Last 3 Years that Used the Tool (Author/Year)
Assessment of Mother-Infant Sensitivity Scale (AMIS)	Price, 1983 [53]	0–4 months	Mother	Yes	Kachingwe et al., 2021 [64]; Inoue et al., 2022 [50]
BATMAN (Bob and Tom’s method for assessing nutrition)	Klesges et al., 1983 [54]	Pre-school age	Mother and Father	Yes	X
Dyadic Interaction Nomenclature for Eating (DINE)	Stark et al., 1995 [55]	from 7 months to 12 years	Mother or Father	Yes	Patton et al., 2020 [29]; Garcia et al., 2022 [65]
Feeding Scale; SVIA	Chatoor et al., 1997 [62]; Lucarelli et al., 2002 [63]	0–3 years	Mother or Father	Yes	Cimino et al.,2020 [66]; Mah et al., 2021 [67]; Chatoor et al., 2022 [68]; Cimino & Cerniglia 2022 [51]; Cerniglia et al., 2022 [69]; Pascale et al., 2022 [70]; Cimino, Tambelli, Di Vito, D’Angeli, & Cerniglia, 2022 [71]; Cimino, Almenara, & Cerniglia, 2022 [28]
Lausanne Trilogue Play (LTP)	Fivaz-Depeursinge & Corboz-Warnery, 1999 [61]	2–17 years	Mother and Father	Yes	Criscuolo, Laghi, Mazzoni, Castiglioni, Vicari, & Zanna, 2020 [72]; Mensi et al., 2020 [73]; Criscuolo, Zanna, Mazzoni,& Laghi, 2020 [74]; Witte et al., 2020 [75]; Foddis et al., 2021 [76]; Liang et al., 2021 [77]; Favez et al., 2021 [78]
Mealtime Interaction Coding System (MICS)	Hayden et al., 1998 [60]	from 12 months to 13 years	Mother and Father	Yes	Pesch et al., 2020 [40]; Smith et al., 2022 [79]
Mealtime Observation Schedule (MOS)	Sanders, Patel, Le Grice, & Shepherd, 1993 [56]	a wide age range (including 0–3 years)	Mother or Father	Yes	X
Mother-Infant Feeding Tool (MIFT)	Brown, Thoyre, Pridham, & Schubert, 2009 [52]	1–4 months	Mother	Yes	X
Nursing Child Assessment Satellite Teaching (NCAST)	Barnard, 1978 [57]	0–36 months	Mother or Father	Yes	Church et al., 2020 [80]; Leung et al., 2020 [81]; Olds et al., 2020 [82]; Alhusen et al., 2021 [83]
Parent Child Early Relational Assessment (PCERA)	Clark, 1985 [58]	0–5 years	Mother or Father	Yes	X
Responsiveness to Child Feeding Cues Scale (RCFCS)	Hodges, Johnson, Hughes, Hopkinson, Butte, & Fisher, 2013 [59]	0–2 years	Mother or Father	Yes	Black et al., 2022 [84]

## 4. Discussion

The role of the caregiver is crucial. According to Bowlby [85], a successful caregiver contributes to the proper emotional, cognitive, and psychosocial development of children. Identifying and understanding how certain characteristics of caregiver-child interaction may put children at psychological risk in the future is of enormous value. For this reason, increased assessment tools have been devised to evaluate several aspects of relationships in the context of interaction quality. Since feeding is considered the most organized and complex behavior in children [86], the study of mealtime interactions will help to understand the processes of regulating caregiver-child interactions. In fact, the early feeding behaviors of the mother and child are important because they occur frequently and have a regulatory effect on the child’s future development [87]. In addition, interactions that occur during feeding are critical especially for mothers of premature infants [88]. The quality of early feeding interactions can reinforce adaptive or maladaptive feeding behaviors of the mother and baby [52].

The purpose of this review is to identify observational tools for assessing parent-child feeding interactions, to present information to clinicians and researchers in the field, and to find strategies for assessing and observing the dyadic (parent-child) relationship. Because caregiver-child interactions are essential to the child’s later development, assessing the quality of these interactions could be useful in preventing childhood eating disorders and future psychological problems in the child. By observing these moments, both strengths and weaknesses can be identified. In fact, the use of observational tools can be useful to researchers and clinicians who wish to understand both mealtime and relationship quality and how parents and children mutually regulate eating behavior. This review will help researchers more quickly identify which tool to use in cases of early feeding problems in early childhood and analyze the feeding behaviors that can make a difference in the adaptability of the feeding process and feeding outcomes. Indeed, through early assessment of parent and child adaptive processes that occur in real-time during feeding interaction, it will be possible to address critical areas for caregiver-child dyads.

One aspect to consider is the need for specific training to use the instruments identified in this review. Indeed, to obtain a valid observation, and thus to score it in a way that reflects what was observed during the recording, it is important to have appropriate training and to pass a reliability test. Because caregiver-child relationships and interactions during feeding are complex to analyze and interpret, it is necessary for specialists to be knowledgeable about them and to conduct reliable assessment procedures. All scales examined in this review (AIMS, BATMAN, DINE, FEEDING SCALE, SVIA, MOS, MIFT, NCAST, PCERA, RCFCS, MICS, LTP) require specific and/or certified training by the coder. This reinforces the veracity and reliability of the results, although training procedures can sometimes be expensive and time-consuming.

Comparing the observational power tools on training shows the following: (1) The AMIS tool needs training and evaluation criteria to provide a basis for training potential users. (2) In the BATMAN instrument, observers are taught the operational definitions of behavioral categories and general procedures for recording data; coders practice coding recorded videotapes until they reach an agreement of at least 0.90. (3) DINE coders were trained on the DINE using the procedures established by the authors themselves until they independently achieved 70% reliability on the videotape. (4) Coders on the power scale and SVIA periodically conduct a training course on the application of the instrument in normal and clinical populations. The course includes an evaluation of observer reliability against the Scale coding system. (5) Coders have been trained in the MOS over the course of several sessions and meet periodically during coding to minimize errors; two experienced observers trained in the use of the instrument code video recordings of parent-child interaction, and a third observer verifies the reliability. (6) All videotapes were coded by one MIFT-trained coder; a second coder trained to 80% reliability coded a random 20% of the videotapes. (7) To be qualified to perform NCAST scoring, attendance at a workshop conducted by an NCAST-certified instructor is mandatory; inter-observer reliability of 85% must be achieved to use the scales in clinical work and 90% for use in research. (8) An observer trained on the PCERA explains to others how to score according to the PCERA manual. (9) RCFCS coders receive training for a period of 2 weeks before coding begins and are required to go through training to code 85% of gold standard videos; usually the training lasts 3 months, and coders meet during the week to address and solve problems that arise during the coding process. (10) Coding for MICS is performed by trained independent coders who are blind to the study hypotheses. (11) Finally, coders are also required to perform training when evaluating videos according to the LTP scale.

### 4.1. An Online Observation on the Assessment of Feeding Interactions: The SVIA

The SVIA [63], an Italian version of the Feeding scale [62], is used on children aged 1 to 36 months. It assesses interactive behaviors and identifies typical and/or dysfunctional relational patterns in parent-child feeding exchanges. Parent-child interactions during mealtime are recorded for 20 min, and mother-child interactive behaviors are analyzed and evaluated by two experienced practitioners. As we mentioned, the SVIA consists of 41 items, divided into 4 subscales that include (1) Parent’s affective state, which indicates the parent’s affective states (e.g., the parent appears sad during feeding); (2) Interactive conflict, which indicates interactions characterized by non-empathic, conflicting and non-cooperative communication (e.g., the parent forces food into the child’s mouth); (3) Food refusal behavior, habits associated with difficult regulation during feeding; and (4) Affective status of the dyad, indicating the extent to which the child’s feeding patterns are or are not the result of interactive regulation to which both partners contribute (e.g., parent and child show joy during feeding). Scores are measured on a Likert scale ranging from 0 (none) to 4 (very much). Higher scores indicate more dysfunctional interactive patterns. An overall score can be used to differentiate adaptive from maladaptive parent-child interaction by considering the sum of the four scores.

The SVIA has been used for more than 20 years to assess the quality of dyadic exchanges between parents and children during feeding. Recently, during the COVID-19 pandemic, it began to be administered online, through the use of digital tools, and the results were positive overall. In the study conducted during the COVID-19 pandemic [51], the authors describe that parents and children experienced remote administration of SVIA and the rate of family participation was high. The longitudinal study shows that allowing the family to have direct (albeit remote) contact with clinicians improved the quality of dyadic interactions during feeding. In addition, the active participation of parents was demonstrated.

It would be useful in the future to experiment with using the other tools described above remotely, to have greater flexibility in their use and to extend the possibility of participation to families unable to move.

### 4.2. Limitations and Suggestions for Future Research

This review has some limitations. First, it is not a systematic review, so it does not follow a systematic approach. However, we believe it can be useful as it describes observational tools used through videotaping and thus potentially usable at a distance. Second, our focus has been only on child-parent(s) interactions, but it is worth noting that there are several reference figures such as parties, relatives, and siblings. Moreover, another limitation is related to the age group: it would be interesting to investigate observational-type instruments for school-age children but also adolescents.

Future studies could investigate these limitations and also evaluate the differences between online versus live observation on the assessment situation, in fact, it is possible that observation of parents’ interaction with their children would yield different results in an online context than in a live observation situation because the people being observed might be influenced by the camera differently than a live observer [89].

## 5. Conclusions

Studies conducted by Infant Research have explored the characteristics of the caregiver-child relationship, highlighting the skills the child needs to create and maintain relationships and the importance of parenting skills in caregiving. The observational coding system makes it possible to describe the process involved in parent-child feeding interactions and to describe the sequence of parent-child interaction behaviors. Observation of early parent-child interactions, which in the first years of life occur during play or feeding, can help identify and prevent early problems in the dyadic relationship, helping families who may benefit from interventions such as parenting support, before the child’s problem behaviors become established [14,90]. Observation is the best way to assess parent-child behavior. However, few studies use observation scales of remote feeding interactions, with a video recording of the observation made by digital platforms or digital tools. The few available pieces of evidence present overall positive results [51].

In the future, it would be useful to make a comparison between these two methods, (in-person vs. online) so that practitioners and researchers can avoid being influenced by cost or time and have more options according to the needs of families, hospitals, and specific economic and social situations, choosing the most appropriate observational method to obtain concrete and satisfactory results.

## Figures and Tables

**Table 1:** Keywords.

Infan	Observational tool	Technology	Meal
Child	Assessment	Computer methodolog	Feeding
Preschool	Measure	Video	“eating disorder”
	Instrument	Telehealth	“eating problem”

**Table 2:** Selected studies.

Title	Author/Year	Tools Employed
A Study on Online Intervention for Early Childhood Eating Disorders during COVID-19	Cimino, Almenara, & Cerniglia, 2022 [28]	Scala di Valutazione delle Interazioni Alimentari (SVIA)
Associations between autism symptom severity and mealtime behaviors in young children presented with an unfamiliar food	Patton et al., 2020 [29]	Dyadic Interaction Nomenclature for Eating (DINE)
Behavioral and Psychophysiological Responsiveness During Child Feeding in Mothers with Histories of Eating Disorders: A Pilot Study	Hoffman et al., 2013 [30]	Responsiveness to Child Feeding Cues Scale (RCFCS)
Development of the General Parenting Observational Scale to assess parenting during family meals	Rhee, Dickstein, Jelalian, Boutelle, Seifer, & Wing, 2015 [31]	Mealtime Family Interaction Coding System (MICS)
Effect of early skin-to-skin contact on mother–preterm infant interaction through 18 months: Randomized controlled trial	Chiu & Anderson, 2009 [32]	Nursing Child Assessment Satellite Training (NCAST; Feeding Scale and Teaching Scale)
Effects of Early Parent Training on Mother-Infant Feeding Interactions	Globus, Latzer, Pshetatzki, Levi, Shaoul, Elad, & Rozen, 2019 [33]	Feeding Scale
Excessively crying infant in the family: mother-infant, father–infant and mother–father interaction	Räihä, Lehtonen, Huhtala, Saleva, & Korvenranta, 2002 [34]	Parent–Child Early Relational Assessment scale (PCERA)
Exploration of Responsive Feeding During Breastfeeding Versus Bottle Feeding of Human Milk: A Within-Subject Pilot Study	Whitfield & Ventura, 2019 [35]	Nursing Child Assessment Satellite Training (NCAST; Feeding Scale and Teaching Scale)
Exploring Correlates of Infant Clarity of Cues During Early Feeding Interactions	Ventura, Sheeper, & Levy, 2019 [36]	Nursing Child Assessment Feeding Scale (NCAFS)
Family-Based Intervention to Enhance Infant–Parent Relationships in the Neonatal Intensive Care Unit	Browne & Talmi, 2005 [37]	Nursing Child Assessment Feeding Scale (NCAFS)
Fathers’ attachment representations and infant feeding practices	Reisz, Aviles, Messina, Duschinsky, Jacobvitz, & Hazen, 2019 [38]	Feeding Scale
Feeding Development, Father Involvement and Family Interactions: Comparison of Two Single-Cases	Hall, Simonelli, & Viola, 2014 [39]	Feeding Lausanne Trilogue Play
Feeding Styles among Mothers of Low-Income Children Identified Using a Person-Centered Multi-Method Approach	Pesch et al., 2020 [40]	Mealtime Family Interaction Coding System (MICS)
Infantile Anorexia and Co-parenting: A Pilot Study on Mother-Father-Child Triadic Interactions during Feeding and Play	Lucarelli, Ammaniti, Porreca, & Simonelli, 2017 [41]	Lausanne Trilogue Play
Iron-deficiency anemia (IDA) in infancy and mother-infant interaction during feeding	Armony-Sivan, Kaplan-Estrin, Jacobson, & Lozoff, 2010 [42]	Nursing Child Assessment Feeding Scale (NCAFS)
Malnutrition and Dysfunctional Mother-Child Feeding Interactions: Clinical Assessment and Research Implications	Ammaniti, Ambruzzi, Lucarelli, Cimino, & D’Olimpio, 2004 [43]	Scala di Valutazione delle Interazioni Alimentari (SVIA)
Mindless feeding: Is maternal distraction during bottle-feeding associated with overfeeding?	Golen & Ventura, 2015 [44]	Nursing Child Assessment Feeding Scale (NCAFS)
Mother–child feeding interactions in children with and without weight faltering; nested case control study	Robertson, Puckering, Parkinson, Corlett, & Wright, 2011 [45]	Simplified version of the Mellow Parenting Coding System (MPCS)
My Child at mealtime parent self-assessment of food related behaviors: Validation with mealtime behaviors	Ontai, Sutter, Sitnick, Shilts, & Townsend, 2019 [46]	Adapted from the modified BATMAN (Bob and Tom’s Method for Assessing Nutrition)
Parent-infant interaction in the NICU: Challenges in measurement	Richter, Fehringer, Smith, & Pineda, 2022 [47]	Nursing Child Assessment Feeding Scale (NCAFS)
Perceived and observed parenting behavior in American and Italian first-time mothers across the first 3 months	Hsu & Lavelli, 2005 [48]	Assessment of Mother–Infant Sensitivity Scale (AMIS)
Preterm birth and Assisted Reproductive Technology/ART: Maternal emotional wellbeing and quality of mother-newborn interaction during the first three months of life	Tallandini, Morsan, & Macagno, 2012 [49]	Nursing Child Assessment Feeding Scale (NCAFS)
Smartphone use during breastfeeding and its impact on mother–infant interaction and maternal responsiveness: Within-subject design	Inoue, Hashimoto, Nakatani, & Ohira, 2022 [50]	Assessment of Mother–Infant Sensitivity (AMIS)
The Effect of a Telehealth Intervention on Mother–Child’s Feeding Interactions During the COVID-19 Pandemic	Cimino & Cerniglia, 2022 [51]	Scala di Valutazione delle Interazioni Alimentari (SVIA)
The Mother-Infant Feeding Tool	Brown, Thoyre, Pridham, & Schubert, 2009 [52]	Mother-Infant Feeding Tool

## Data Availability

Not applicable.
